# Benefits of Chain-of-Thought Prompting for Clinical Record Rubric Evaluation in Undergraduate Medical Education: Experimental Evaluation Study With Medical Faculty

**DOI:** 10.2196/88652

**Published:** 2026-07-23

**Authors:** Alberto Nogales, Sophia Denizon, Alonso Mateos Rodriguez, Javier Cervera Cordero, Gonzalo Pandelet Barainca, Enrique Aranguren Moliner, Emilio Cervera Barba

**Affiliations:** 1 CEIEC (Centro de Innovación Experimental del Conocimiento), Universidad Francisco de Vitoria, Pozuelo de Alarcón, Madrid, Spain; 2 Department of Computer Science, University of Alcalá, , Alcalá de Henares, Spain; 3 Centre for Advanced Clinical Simulation, Faculty of Medicine, Universidad Francisco de Vitoria, Ctra. Pozuelo-Majadahonda, km 1,800, Pozuelo de Alarcón, Madrid, 28223, Spain, 34 917091400 ext 1670, 34 917091555

**Keywords:** medical records, medical students, large language models, chain-of-thought, formative feedback, clinical competence

## Abstract

**Background:**

Large language models in artificial intelligence have been among the tools with a significant and real impact on people’s daily lives. In this regard, they serve as an aid in specific fields, such as education, helping educators with cumbersome tasks such as periodic evaluations.

**Objective:**

This study focused on analyzing the benefits of large language models, particularly the chain-of-thought (CoT) strategy, for the task of evaluating students’ Spanish-language medical record writing. The aim was 2-fold: first, we attempted to save time and resources, and second, we used the reasoning of the CoT strategy to evaluate the rubrics and their interpretations.

**Methods:**

The proposed solution assessed the application of 2 models—Llama 3.1 and Claude 3.5—in combination with one-shot and CoT to evaluate how medical students write medical records in Spanish. First, machine learning metrics were applied to measure the performance of the solutions. Then, different statistical analyses were performed at the clinical record and item levels. Finally, differences between the proposed models and evaluators were studied in depth.

**Results:**

A maximum of 3807 items were evaluated. Claude obtained the best accuracy with slight differences between one-shot and CoT (86.4% and 85.0%, respectively). However, Claude with CoT outperformed the rest of the combinations on all complementary metrics, initially achieving a sensitivity of 94.2%, specificity of 59.5%, precision of 85.8%, and *F*_1_-score of 89.6%. Expert review of CoT reasoning determined that 63.8% of the discrepancies were model hits, raising Claude’s final accuracy to 94.6% (SD 4.3%). In the final phase, sensitivity was 98.0% (SD 2.3%), specificity improved to 83.3% (SD 14.3%), and *F*_1_-score reached 96.2% (SD 3.3%). Sectional analysis showed greater difficulties in the “History of present illness” section (n=125 discordances).

**Conclusions:**

CoT demonstrated strong potential for supporting the evaluation of clinical records written in Spanish by medical students and providing feedback to them. More importantly, it showed significant promise in assisting professors by assessing the quality of their rubrics and identifying possible errors.

## Introduction

### Background

The early acquisition of skills in clinical record writing is a fundamental learning objective in medical education, given its central role in both clinical care and training. The medical record not only documents the patient’s encounter but also initiates and structures clinical reasoning. It provides the narrative framework through which students organize collected data, prioritize problems, identify uncertainties, and articulate hypotheses and differential diagnoses. Evidence from the literature indicates that a substantial proportion of diagnostic accuracy stems from the quality of data gathered from the patient and the proper synthesis of the data, often surpassing the contribution of physical examinations or complementary tests [1-3].

Furthermore, the process of gathering patient information constitutes the foundation of the clinical relationship. Established educational frameworks, such as the Calgary-Cambridge Guides, highlight the communication competencies required to elicit accurate information while incorporating the patient’s perspective. Embedding these elements within the medical record supports comprehensive and patient-centered care [4].

In addition, the medical record functions as the official register of care. It documents patient data, clinical decisions, diagnostic and therapeutic processes, and informed consent, making it indispensable for patient safety, quality assurance, care management, and the ongoing learning of professionals and health care organizations. At the same time, it underpins the continuity of care by facilitating the transfer of information across professionals and levels of the health care system [5].

Beyond its clinical and organizational functions, the medical record also carries significant legal weight. As the principal medicolegal document, its completeness and accuracy are critical in ensuring accountability and may prove decisive in the event of malpractice litigation [6,7].

Despite the central role of the medical record, clinical clerkships do not always guarantee that medical students acquire solid competencies in collecting and writing medical histories. The learning experience is highly dependent on prior instruction, the dynamics of the health care team, the presence of junior doctors, and, critically, the lack of structured feedback [8]. Previous studies have reported significant variability in the quality and completeness of medical students’ clinical documentation, emphasizing the need for structured evaluation methods and clearer assessment frameworks [9].

To address these limitations, simulated patient encounters have become a highly valuable and standardized approach in medical education for teaching and assessing clinical skills. They enable the evaluation of how students collect patient data, prioritize problems, and justify diagnostic decisions, while also offering the additional benefit of structured feedback from the simulated patients themselves [10]. Building on this, structured feedback programs have been shown to significantly improve students’ competence and preparedness [11-13]. However, providing consistent and timely feedback on free-text clinical records is highly demanding for faculty and raises the challenge of reliability between educators [14]. Moreover, assessment tools, such as rubrics, can introduce variability between evaluators if their criteria are not carefully defined and validated, which highlights the importance of methodological calibration in educational assessment [15].

In this context, artificial intelligence (AI), and particularly large language models (LLMs), have emerged as promising tools in medical education. The World Health Organization has highlighted the growing need to integrate AI training within medical education, acknowledging that AI technologies, spanning from diagnostic algorithms to clinical decision-support systems, are progressively being adopted in modern clinical practice. Applications include medical school admissions, innovative teaching and learning methodologies, the evaluation of clinical records and manual skills, clinical simulation, and the generation of formative feedback at scale [16,17].

Previous systematic reviews have tried to measure the impact of educational interventions in improving and maintaining the quality of medical records. A previous study found that interventions most frequently consisted of traditional educational approaches, such as lectures, feedback sessions, workshops, and group discussions, addressing different components of clinical documentation [18]. Overall, these interventions showed positive effects on participant satisfaction as well as improvements in documentation skills and attitudes toward record-keeping. However, the methodological quality of the evidence was limited, highlighting the need for more rigorous study designs.

In recent years, AI and LLMs have been widely applied in the field of medical education, having a significant impact as highlighted below. The time and resource savings associated with the use of LLMs for evaluating student records, along with their high effectiveness and agreement with human evaluators, have already been demonstrated in previous studies [19-23]. The review by Wei et al [19] is a systematic review focused on assessing ChatGPT’s performance in answering medical questions. The authors concluded that there are inconsistencies that highlight important limitations in the current evidence base and emphasized the need for standardized evaluation frameworks and more transparent reporting when assessing the performance of LLMs in medical question answering.

In the specific context of medical history taking, a study aimed to assess whether LLMs could generate valid clinical interview dialogues to support medical education [20]. A fine-tuned Gemma-3-27B model was compared with GPT-4o-mini in simulated chest pain interviews, with Claude 3.5 Sonnet inferring diagnoses. The results, which were evaluated using a chest pain checklist, were promising.

Another study investigated whether ChatGPT could match junior medical residents in history taking during simulated patient encounters [21]. The findings indicated that ChatGPT-4 achieved comparable performance in information gathering but was weaker in communication and empathy.

Several studies have also compared LLM-based assessments with those performed by human evaluators. One study contrasted student record evaluations conducted by standardized patients versus GPT-3.5 [22]. Standardized patients made errors in 7.2% of the items, whereas the error rate of GPT was only 1%. Another study reported 83% to 90% agreement between AI models and human evaluators, reducing manual grading workload by up to 91% [23].

### Goal of This Study

This study investigated the potential benefits of chain-of-thought (CoT) prompting for the assessment of medical records in undergraduate medical education. Specifically, we compared CoT prompting with a standard one-shot prompting strategy, which serves as a baseline approach commonly used in LLM applications. The study focuses on their agreement with human evaluators and their potential for correcting rubrics and providing formative feedback. The study builds upon the limitations of a previous study by Maitin et al [24], which reported superior performance of low-rank adaptation (LoRA) for the proposed task but offered no additional analysis beyond performance results. In this context, the study seeks to leverage the benefits of CoT prompting, which enhances reasoning in complex tasks, generalizes across domains and languages, and provides interpretable step-by-step rationales [25].

The main research question of this study is whether LLMs, and specifically CoT prompting, can support medical instructors in the evaluation of clinical records. We studied whether CoT prompting can facilitate the assessment process by generating structured explanations that may help accelerate the grading workflow and promote greater consistency in evaluations. More importantly, we explored whether the reasoning traces produced through CoT prompting allow instructors to analyze and refine the precision of their evaluation criteria by making the reasoning steps underlying the automated assessment explicit.

## Methods

### Study Context and Rubric Design

During their medical degree, students train in communication and clinical interview skills through scenarios with simulated patients. Following each encounter, they are required to produce a complete medical record, submitted as a Word document (Microsoft Corp) via the virtual campus. Faculty members then correct these records and assess performance using a standardized 48 item yes/no rubric, which covers the following: patient identification and chief complaint (2 items), medical history (14 items), history of the present illness (21 items), physical examination (7 items), assessment and plan (3 items), and order and clarity of writing (1 item). An example of the rubric is provided in Multimedia Appendix 1.

The 48-item rubric used in this study is a standardized tool that has been used in the Faculty of Medicine at Universidad Francisco de Vitoria for at least 6 academic years (2020‐2026) to evaluate clinical records written following simulated patient encounters. This rubric was originally developed by the medical teaching staff based on international clinical guidelines, forensic requirements for clinical documentation in Spain, and the specific competencies of the curriculum. The rubric has been used in previous studies by the same faculty members who established its interrater reliability [26].

In this study, a set of clinical histories is analyzed both entirely and at the item level of the rubric, using one-shot and CoT prompting strategies in the following two LLMs: (1) Llama 3.1 70B [27] and (2) Claude 3.5 Sonnet [28].

### Study Design

**Figure 1.**
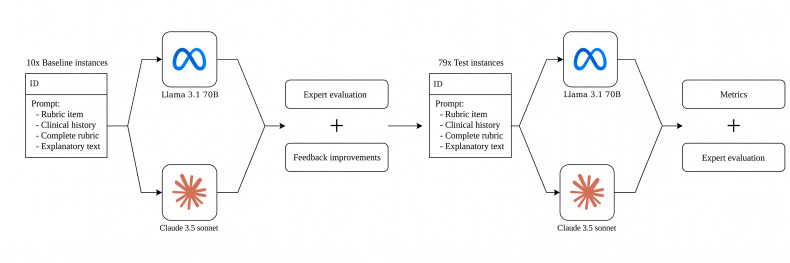
Workflow followed in this study.

As the aim of this study was to evaluate the benefits of using CoT in the context of helping university professors to correct clinical records written in Spanish by degree students, the workflow depicted in Figure 1 was implemented.

First, we evaluated 10 preliminary clinical records corrected by Llama and Claude using one-shot and CoT. The results were reviewed by professors in medicine to improve the rubric used for the final evaluation. This process was carried out not to validate the rubric per se, but rather to verify that the LLMs correctly interpreted each item when the prompting strategy was applied, particularly in Spanish, and that there were no semantic ambiguities that could generate systematic divergences between human and AI evaluations. It is important to note that these refinements were applied to the prompt design and did not modify the conceptual content of the evaluation rubric. During this preliminary phase, minor adjustments were identified in the wording of some items to improve clarity. For example, it was specified that the mention of a clinical concept (eg, headache) should be recorded as “Yes” regardless of whether it was present or explicitly denied in the clinical record (active negation), rather than only when the simulated patient actually experienced the symptom. These clarifications were incorporated into the final prompt used in the study.

**Figure 2.**
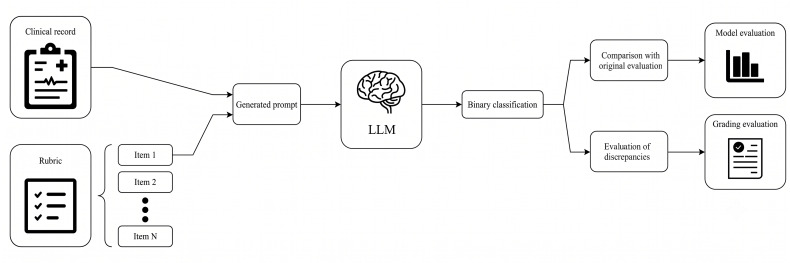
Diagram flow of the 2-fold evaluation. LLM: large language model.

Following this preliminary phase, we proceeded with the evaluation of the clinical records constituting the main corpus of this study. In this second stage, 79 clinical records were again processed by Llama and Claude using one-shot and CoT. Clinical records were selected based on faculty judgment to combine records from different pathologies in which students had trained during the course and to include varying levels of initial record quality, thereby promoting variability in the analysis by the models. The quality of the results was evaluated using AI metrics, such as accuracy, sensitivity, and specificity, in 2 complementary ways. Figure 2 describes both evaluations.

First, the accuracy of the LLM-generated evaluations was assessed by comparing the item responses produced by the models with the reference evaluations assigned by the professors when grading the test. This comparison allowed us to determine which prompting strategy and LLM configuration achieved the best performance.

**Figure 3.**
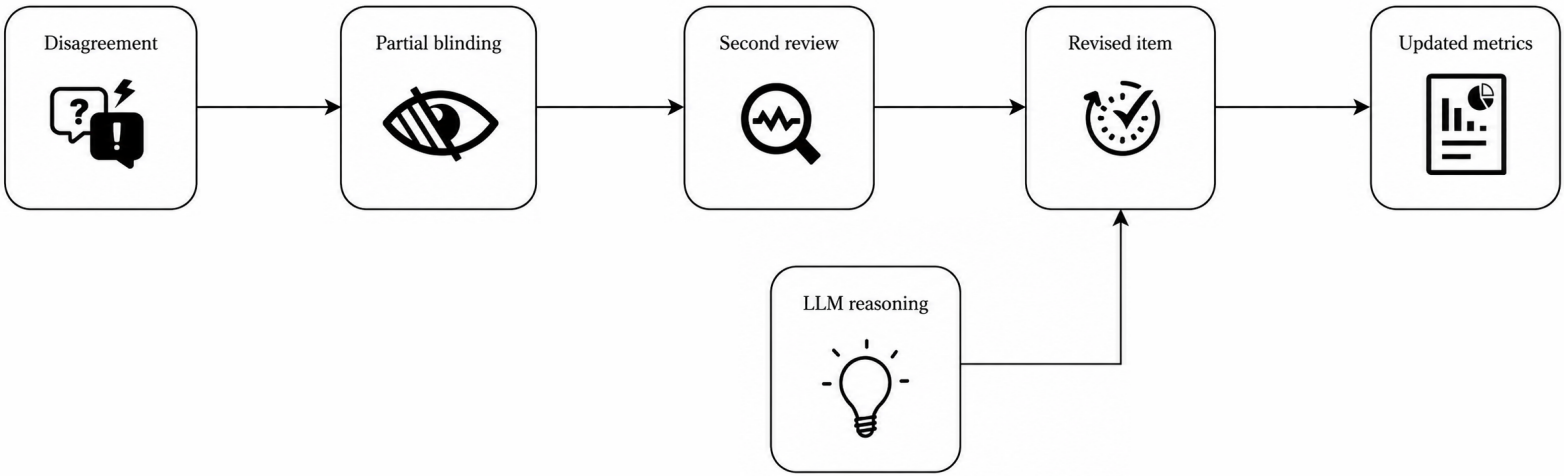
Diagram for the adjudication process. LLM: large language model.

Second, we performed a qualitative analysis of the cases where discrepancies were identified between the responses of the best-performing model using CoT and the professors’ evaluations. These disagreements were reviewed by human experts to understand whether they resulted from model errors, ambiguities in the evaluation rubric, or differences in interpretation during grading. In this process, 3 medical professors with more than 15 years of experience in clinical teaching, with demonstrated high interrater concordance, independently reviewed the disagreements. To mitigate confirmation bias in the adjudication process, which could confer greater perceived authority to the model’s judgment over human evaluation, several methodological safeguards were implemented as follows: (1) partial blinding was implemented, whereby adjudicators reanalyzed each item independently; (2) one of the professors conducted a second review in cases where disagreement persisted; and (3) the model’s reasoning was examined only to inform the final decision. This analysis provided additional insights into how the evaluation criteria are applied and whether LLMs can help instructors reflect on the consistency of their grading practices. The adjudication process is depicted in Figure 3.

### Ethical Considerations

This study was conducted in accordance with the ethical principles outlined in the Declaration of Helsinki and adhered to the good clinical practices established in Spanish legislation (Law 14/2007 on Biomedical Research [29]). The data contributed by participating students, obtained from their clinical record submissions, were handled confidentially in compliance with current Spanish regulations (Organic Law 3/2018 on personal data protection and the guarantee of digital rights [30]). All records were pseudonymized at the source through the assignment of a unique alphanumeric code to each student. The research team committed to refraining from any action that could lead to participant identification, thereby ensuring full anonymity. The study was carried out with full respect for the participants’ fundamental rights and freedoms. Importantly, the results obtained in this study were used exclusively for research purposes and had no influence whatsoever on the participants’ academic grading. Ethical approval was implicit, as the project received a research grant awarded by Universidad Francisco de Vitoria.

### Data Source

As mentioned earlier in the text, the final aim of the study was the evaluation of the writing in clinical records using different rubrics that were dependent on the medical use case. In this way, the final evaluation consisted of 79 medical records written in Spanish from standardized patient encounters covering cases of polymyalgia rheumatica, tensional headache, acute cholecystitis, and mechanical chest pain. A request made to the LLM consisted of an item of the rubric to be evaluated in the clinical record. The correction rubrics for the cases consisted of 48 items, except for the rubric on headache, which included 49 items owing to the addition of an item related to physical examination. Each rubric item was scored as either correct or not correct. The label distribution was binary and dominated by positive labels, with a “Yes” rate between 52% and 78% depending on the case type.

### Models and Strategies

The following text defines the concepts and methods related to models and strategies used in this study.

Llama 3.1 70B Instruct is a transformer-based decoder-only language model developed by Meta. Released on July 23, 2024, it comprises 70 billion parameters and has been trained on a multilingual, multimodal dataset spanning roughly 15 trillion tokens [31]. The model was accessed through the Hugging Face repository [27]. It should be noted that no parameter updates affecting inference were reported after the initial July 2024 release. The model was configured with a temperature of 0.6, top-p of 0.9, and maximum output length of 512 tokens.

Claude 3.5 Sonnet, developed by Anthropic, is an LLM with approximately 175 billion parameters and a context window of 200,000 tokens. It was designed with a strong emphasis on safety and ethical considerations, and reports indicate that it outperforms GPT-4o and Gemini 1.5 Pro across multiple domains. For our study, we specifically used the updated version of Claude 3.5 Sonnet, released on October 22, 2024 [32]. Although this version has sometimes been informally referred to in the community as Claude 3.6, the official application programming interface (API) identifier remains Claude 3.5 Sonnet (v2). The model was configured with a temperature of 1.0, top-p of 1.0, and maximum output length of 1024 tokens.

According to Brown et al [33], one-shot prompting is the setting where an LLM is conditioned on a single demonstration of the task (an example of input and correct output) before being asked to perform the task on new instances.

CoT is a prompting strategy that guides LLMs to articulate their intermediate reasoning processes before generating the final response [34]. In clinical tasks, where the text is often lengthy, heterogeneous, and rich in nuances (synonyms, abbreviations, negations, or indirect references), this explicit guide directs the model to locate, contrast, and substantiate the presence of a concept in the document, promoting more accurate and justified responses. While its use has recently expanded with the emergence of reasoning models, at the time of the study, none were available, and thus, we used a nonreasoning model instead.

### Scoring Criteria

We formalized the metrics used to evaluate the corrections generated by the LLMs. For this purpose, we compared the item-by-item agreement between faculty evaluations and LLM outputs. True positive (TP) corresponds to a correct answer by the student, which is also classified as correct by the LLM, while true negative (TN) refers to an incorrect student response that the LLM likewise identifies as incorrect.

Accuracy has been used as an initial way to measure the performance of a model when determining whether a response is correct or incorrect. However, in cases of unbalanced datasets and in domains, such as medical education, it is essential to examine not only overall accuracy but also how the model fails, by reporting false positives (FPs) and false negatives (FNs) through complementary metrics such as specificity and sensitivity. Specificity measures the impact of correct answers that were mistakenly flagged as wrong. This metric is particularly valuable to avoid overpenalizing students by incorrectly classifying correct responses as errors. On the other hand, sensitivity reflects the model’s ability to minimize cases where a correct answer is misclassified as wrong. In educational settings, high sensitivity is critical to ensure that the valid knowledge of students is recognized and not overlooked.

In addition, precision and *F*_1_-score provide complementary perspectives on model performance. Precision measures the proportion of answers flagged as incorrect that are indeed incorrect, thus reflecting the reliability of the model when it assigns an error label. High precision is important to maintain trust in the system’s feedback during the evaluation. The *F*_1_-score, defined as the harmonic mean of precision and sensitivity, offers a balanced evaluation by jointly considering FPs and FNs. This metric is especially useful in educational contexts where both correctly identifying student errors and avoiding misclassification of correct answers are equally important.

### Prompting Design

Considering that CoT is being used for the evaluation, there is a need to design specific prompts for the task of correcting clinical records written in Spanish by medical students. This process is described as follows. As mentioned earlier, the study evaluated 3582 requests (1 per rubric item to evaluate and consider API errors), which followed the same prompt structure, with certain elements adapted depending on the clinical record.

The requests are processed as follows. We used 4 rubric categories, each containing a set of items. They all shared the same items (48 items), except the rubric for headache, which had an extra item. Each item consisted of a name and a brief explanation (eg, “Name and age: The medical record contains the patient’s name and age”). For each medical record, these items were systematically evaluated (E indicating evaluation):

- E.1. Select the corresponding rubric (polymyalgia rheumatica, headache, cholecystitis, or chest pain).
- E.2. Iterate through the items in the rubric.
- E.3. For each item, construct a prompt.
- E.4. The model analyzes the record following guided steps (CoT) and issues a binary conclusion: Y (mentioned)/N (not mentioned).
- E.5. The responses are aggregated to obtain metrics per item, per record, and overall.

Considering the workflow depicted above, a total of 3582 effective requests were made to both LLMs following a CoT strategy. Since the rubric’s clinical records correspond to 4 different case types, the prompts, while following a common structure, varied in several elements designed to support reasoning. Each request had the prompting structure as follows (P indicates prompt):

- P.1. Task to accomplish. Determine whether a specific medical concept (item) appears in a clinical record.
- P.2. Item to evaluate. The target concept is set.
- P.3. Step-by-step analysis (4 checks)
  - Detect direct mentions of the exact concept.
  - Identify variations: technical synonyms, colloquial terms, abbreviations/acronyms, and grammatical variants.
  - Classify context: affirmative, negative/discarded, or indirect references.
  - Confirm that negations (eg, “does not present,” “no evidence of,” and “is ruled out”) still count as a mention of the concept.
- P.4. Criteria to apply (5 rules):
  - Distinguish between a symptom (reported by the patient) and a sign (observed by the physician during “physical examination”).
  - If the physician does not write anything about the concept, it is not considered a mention.
  - Mentions that refer only to family members do not count, except when they appear explicitly in the section on “family history/F.H.”
  - The concept must appear completely or partially but in a recognizable and exact form (not vague or ambiguous).
  - The conclusion (Yes/No) must be consistent with the detailed analysis.
- P.5. Format of the response. A single block enclosed with some fields, such as direct mentions, synonyms found, reasoning, or conclusion.
- P.6. Attached rubric to guide how the clinical record is evaluated against the relevant items.
- P.7. Clinical texts related to the use case. They complement the rubric and apply only to the corresponding rubric points.
- P.8. Clinical record to evaluate and exact item reference.

An example of these prompts is provided in Multimedia Appendix 2.

### Rationale Management

For each request, the models generated a structured XML object containing parameters predefined above, following Anthropic’s recommended prompting format. The content of each field was programmatically extracted using regular expressions. To ensure traceability while preserving deidentification, each request was assigned a deterministic UUIDv5 derived from the case identifier and item number, both at the local and global levels. CoT rationales were therefore systematically linked to specific evaluation instances without incorporating any personally identifiable information. Importantly, these rationales were retained exclusively for auditing and methodological transparency purposes, enabling verification of the model’s reasoning process. In line, the system was not intended to provide full rationale text to students but rather to ensure the explainability of the grading.

## Results

### Distribution of Evaluated Items

The total number of evaluated items was 3807. When Claude was used, this number fell to 3582 due to occasional API request failures that occurred when the batch processing exceeded the usage limits of the API account. These failures generated standard API responses indicating that the usage quota had been temporarily exceeded. The API error affected 225 requests (6.9% of the total). In this regard, the total number of items analyzed was 3582: 446 (12.5%) for cholecystitis, 682 (19.0%) for headache, 1771 (49.4%) for polymyalgia rheumatica, and 683 (19.1%) for chest pain. The distribution of these requests is presented in Table 1.

**Table 1.** Distribution of requests considering use cases.

Cases	Items per rubric, n	“Yes” rate (%), mean (SD)	Medical records, n	Total requests obtained, n	API^a^ errors, n
Cholecystitis	48	52.3 (10.6)	10	446	34
Tensional headache	49	78.5 (11.0)	15	682	53
Polymyalgia rheumatica	48	75.0 (14.5)	39	1771	101
Mechanical chest pain	48	74.6 (11.6)	15	683	37
Total	—^b^	—	79	3582	225

a API: application programming interface.

b Not applicable.

### Overall Performance

To measure the performance of the different models and strategies, we determined accuracy, which served as a guide metric to know if the models were performing well. All details of the different experiments, including combinations of one-shot and CoT with Llama and Claude, alongside mean hits and SDs, are presented in Table 2. These data were obtained by averaging the items per medical record across the 79 records. The total number of evaluated items differed for Claude with CoT due to API errors.

**Table 2.** Accuracy of evaluations by prompting strategy and large language model.

Strategy	Llama 3.1 70B		Claude 3.5 Sonnet	
	Value, n	Accuracy (%), mean (SD)	Value, n	Accuracy (%), mean (SD)
One-shot	3807	78.4 (8.2)	3807	86.4 (8.2)
CoT^a^ before review	3807	79.1 (12.5)	3582	85.0 (8.8)
CoT after review	3807	79.6 (11.4)	3582	94.6 (4.2)

a CoT: chain-of-thought.

Claude with one-shot was the best performer, but it only slightly outperformed Claude with CoT. However, the label review changed this outcome.

Accuracy can be considered a good initial metric, but it is also interesting to measure the impacts of FPs and FNs, as these data could be decisive when choosing a solution. The categorization allowed us to compute sensitivity (the model’s ability to recognize valid corrections), specificity (the model’s ability to reject invalid corrections), precision (the reliability of the model’s predictions), and *F*_1_-score (a balanced measure of the model’s ability to both identify valid corrections and avoid FPs), thereby providing a comprehensive assessment of model performance. In this way, we also calculated the sensitivity, specificity, precision, and *F*_1_-score for the same models and strategies and have compiled them in Table 3. Notably, the total number of items was 3807 across all combinations, except for Claude with CoT, which involved 3582 items.

**Table 3.** Complementary performance metrics by prompting strategy and large language model.

Strategy and model		Sensitivity (%), mean (SD)	Specificity (%), mean (SD)	Precision (%), mean (SD)	*F*_1_-score (%), mean (SD)
One-shot					
Llama 3.1 70B		86.0 (12.1)	49.5 (25.6)	82.0 (13.0)	83.7 (11.8)
Claude 3.5 Sonnet		93.5 (6.3)	65.2 (19.4)	87.8 (8.5)	90.3 (6.4)
CoT^a^ before review					
Llama 3.1 70B		91.6 (11.7)	42.8 (17.8)	80.2 (14.5)	85.1 (12.4)
Claude 3.5 Sonnet		94.2 (5.9)	59.5 (18.2)	85.8 (9.5)	89.6 (6.8)
CoT after review					
Llama 3.1 70B		93.0 (11.2)	55.8 (21.2)	86.3 (13.2)	89.3 (11.5)
Claude 3.5 Sonnet		98.1 (2.2)	83.3 (14.3)	94.6 (5.8)	96.2 (3.3)

a CoT: chain-of-thought.

Based on the findings, Claude with CoT was considered the best option, as it demonstrated superior stability and better complementary metrics.

**Figure 4.**
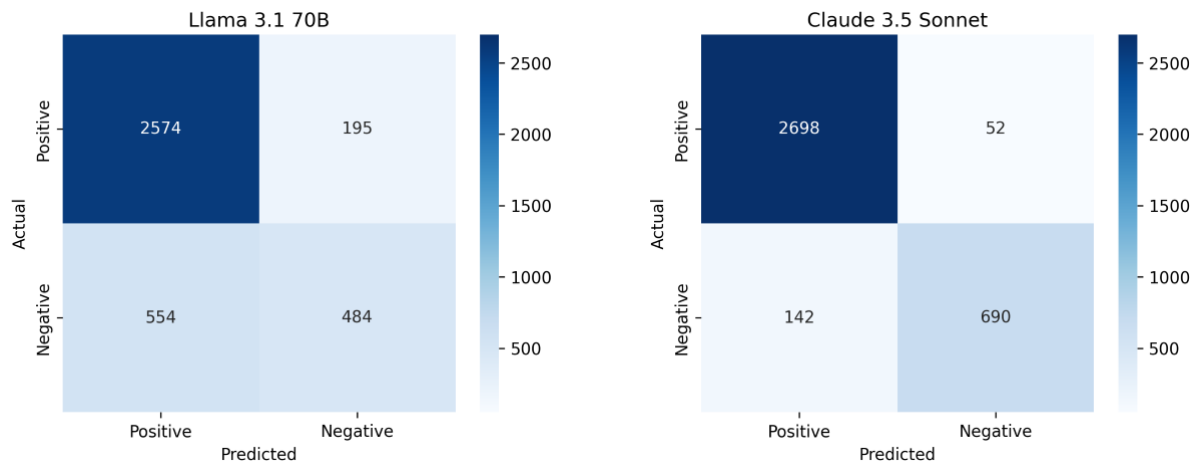
Confusion matrices for chain-of-thought applied to the models.

In addition to reporting performance metrics, the inclusion of the confusion matrix provided a more comprehensive and transparent characterization of model behavior. The confusion matrix explicitly presented the counts of FPs and FNs, enabling a direct assessment of error patterns and potential biases in the model’s predictions. Therefore, the confusion matrix served as a complementary tool that enhances interpretability and facilitates a more nuanced evaluation of model performance beyond aggregated metrics. Figure 4 shows the confusion matrix obtained from the previous results.

The confusion matrices provided a detailed assessment of the models’ performance in evaluating test items by distinguishing between valid and invalid responses. The results showed that Claude 3.5 Sonnet achieved higher numbers of both TPs and TNs, along with substantially fewer FNs and FPs, compared to Llama 3.1. This translates to both higher sensitivity, reflecting a stronger ability to recognize valid corrections, and higher specificity, indicating improved rejection of invalid corrections. In contrast, Llama exhibited a noticeable increase in FPs, suggesting a tendency to overestimate item correctness. These findings confirm that Claude provides a more balanced and reliable evaluation.

To further assess the robustness of the reported results, a brief sensitivity analysis for the best model was conducted by stratifying performance metrics according to case type. This approach allowed the evaluation of whether the observed performance was consistent across heterogeneous subsets of data, as different case types may vary in clinical complexity, level of detail, and completeness of documentation. By examining metrics, such as accuracy, sensitivity, and specificity, within each use case, we aimed to identify any systematic variability in model performance that could be masked in the aggregate analysis. The results are shown in Table 4.

**Table 4.** Claude 3.5 Sonnet and chain-of-thought sensitivity check analysis by case type.

Use case	Accuracy (%)	Sensitivity (%)	Specificity (%)
Cholecystitis (n=446)	91.5	97.2	90.8
Tensional headache (n=682)	94.3	97.7	78.7
Polymyalgia rheumatica (n=1771)	95.2	99.0	80.3
Mechanical chest pain (n=683)	95.3	96.6	90.8
Total, mean (SD)	94.0 (1.5)	97.6 (0.8)	85.5 (5.6)

The stratified sensitivity analysis by case type showed that model performance remained consistently high across all clinical scenarios, supporting the robustness of the previous results. Accuracy was stable with a low overall variability (mean 94.0%, SD 1.5%). Sensitivity was uniformly high across all case types, indicating a strong and consistent ability to correctly identify valid items regardless of the clinical context. In contrast, specificity exhibited greater variability, ranging from 78.7% to 90.8%, with notably lower values in tensional headache and polymyalgia rheumatica, suggesting that the model is more prone to FPs in these scenarios. Despite this, the overall specificity remained acceptable (mean 85.5%, SD 5.6%).

### Detailed Analysis at the Clinical Record Level

CoT demonstrated the ability to obtain better results in general, with inconsistencies in the correction of certain items. To address this, we have provided an additional analysis both at the clinical record level and, in greater detail, at the rubric item level.

**Figure 5.**
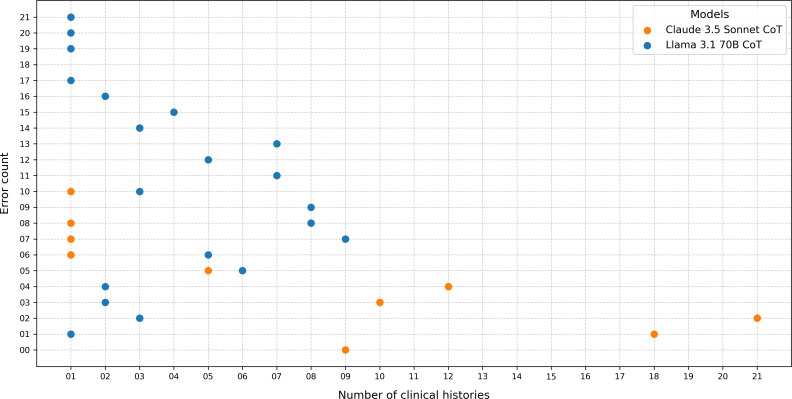
Error counts across the correction of clinical histories using chain-of-thought. Errors are considered discrepancies between the models and evaluators, whether positive or negative. CoT: chain-of-thought.

The first analysis involved measuring whether there were a lot of clinical records with many discordances with respect to the evaluators. To describe this, Figure 5 shows a scatter plot of Llama and Claude using CoT, where a maximum of 21 discordances are noted in 1 medical record and a minimum of 0 discordances are noted in 9 medical records. To establish an adequate academic performance objective, we considered a standard of 70% of the items achieved in each medical record (14 as the maximum number of failed items per history evaluated). This cutoff point coincides with the performance required of medical students in other clinical skills during their practical training.

In both models, most clinical histories presented error counts under 14 items; however, Llama 3.1 exhibited several evaluations with a high number of discordances, reaching between 17 and 21 items in a single record. By examining the Llama errors, it was evident that the model produced a larger number of nonperfectly evaluated clinical histories, although most of them contained only minor errors.

**Figure 6.**
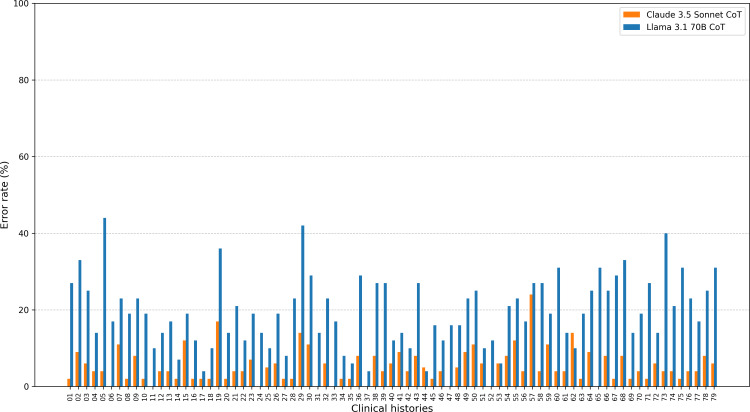
Percentage of disagreements between large language model evaluations and professor evaluations per clinical record. Errors are considered discrepancies between the models and evaluators, whether positive or negative. CoT: chain-of-thought.

To obtain an in-depth perspective on the strategies’ performance, we analyzed clinical histories. Figure 6 shows a comparison of the percentage of discordances for each CoT model. The figure presents the proportion of disagreements between LLM and professor evaluations for each clinical record. The x-axis represents the identifier of each evaluated clinical record, with 2 bars per record corresponding to the 2 used LLMs. The y-axis reports the percentage of rubric items for which the LLM evaluation differs from the score assigned by the professor during grading.

Both models performed reasonably well, but performance occasionally varied depending on the clinical record, and they sometimes systematically triggered a higher rate of disagreement among evaluators, regardless of the model. Claude 3.5 tended to be slightly more stable, avoiding the high values observed with Llama 3.1, which were sometimes 2-3 times higher. This pattern suggests that certain records are inherently more challenging to correct and that both models struggle with them. Furthermore, the analysis highlighted that no single model consistently outperformed the others across all records, although Claude demonstrated slightly better performance by yielding fewer discordances in most of the evaluations.

**Figure 7.**
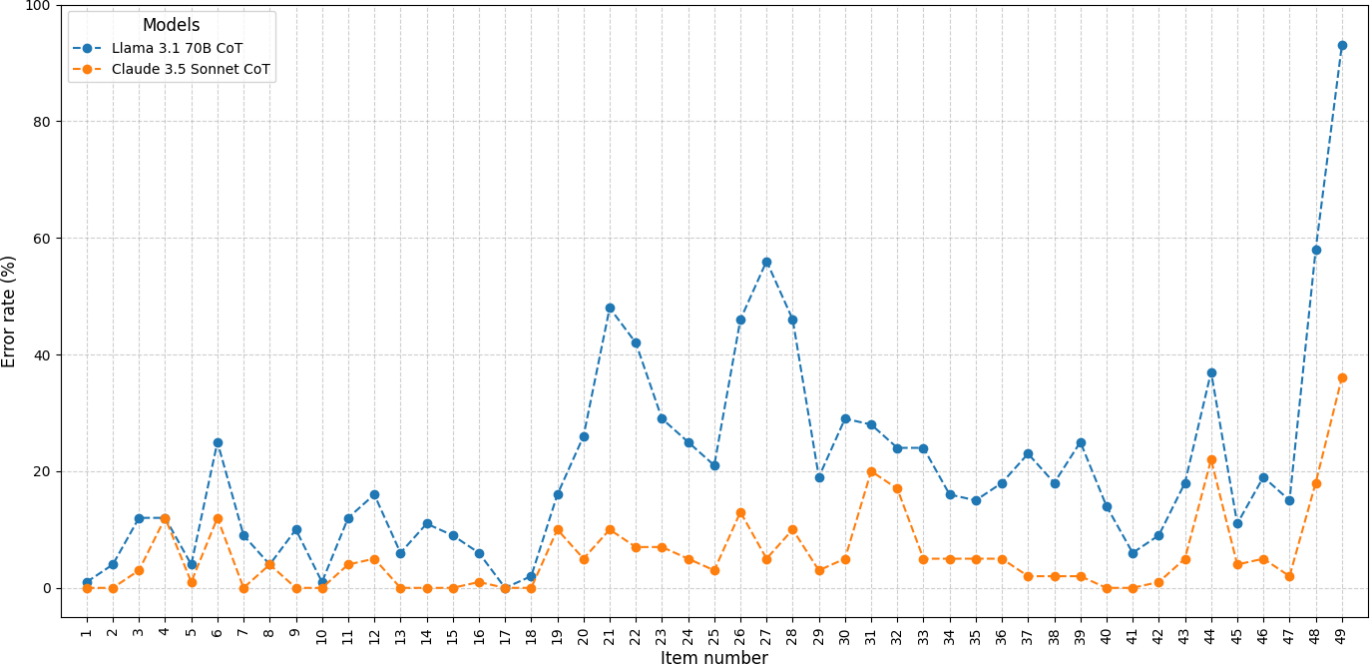
Percentage of disagreements between large language predictions and professor evaluations across rubric items. CoT: chain-of-thought.

A similar comparison has been provided but with consideration of the items of the rubric used by the evaluators. Figure 7 follows the same analysis framework as that in Figure 6 but focuses on the rubric item level, presenting the percentage of disagreements for each of the 49 rubric items using a line plot (1 line per model). The figure aggregates the results across all clinical records. Each point represents the proportion of evaluations in which the model differs from the professor’s score for a given rubric item. The x-axis corresponds to the rubric item identifier, and the y-axis shows the disagreement rate.

Figure 7 illustrates the item-wise error rate distribution for both models across the 49 rubric items. Item 49 was only evaluated in 15 medical records for headache. Both models exhibited heterogeneous behavior, with error rates fluctuating considerably among items, reflecting variable alignment with the reference evaluations.

Overall, Claude 3.5 Sonnet CoT maintained consistently lower error rates throughout the sequence, rarely exceeding 15% or 20%, suggesting greater stability and reliability in its reasoning across diverse rubric items. In contrast, Llama 3.1 70B CoT displayed higher dispersion and more pronounced peaks, with several items surpassing error rates of 30% or 40%, indicating localized weaknesses or inconsistencies in reasoning.

When examining item-level performance in detail, Llama 3.1 70B CoT showed particularly elevated error rates for items 21‐22, 26‐28, 44, and 49, frequently exceeding 40%. For Claude 3.5 Sonnet CoT, only items 31, 44, and 49 surpassed the 20% threshold, with additional moderate deviations observed in items 4, 6, 19, 21, 26, 28, 32, and 48. Notably, item 49 presented an exceptionally high error rate for both systems, although this result should be interpreted cautiously, given its limited sample representation within the dataset.

This pattern suggests that, while both models follow broadly similar trends, Claude’s explicit reasoning process yields more consistent and calibrated judgments, whereas Llama exhibits greater volatility and sensitivity to item-specific complexity.

An analysis of these items from a medical perspective yielded some problems. High error counts were observed in items related to core symptom characterization, including “Symptom characteristics (pressing, stabbing, pulsatile, etc) are described,” “Symptom intensity is recorded,” “Associated symptoms are described,” “Perceived cause of the symptoms is documented,” and “Previous episodes of similar symptoms are recorded.” Additionally, a notable error frequency was noted for the item “Lower extremities (LE): presence or absence of edema, color changes are recorded,” extending these limitations to aspects of the physical examination. These items encompass both fundamental semiological descriptors and basic examination findings, which are typically considered standard components of clinical assessment. The concentration of errors in these domains suggests that the LLMs exhibit difficulties in detecting and assigning specificities of the information collected to the different parts of the medical record. For the item “Lower extremities (LE): presence or absence of edema, color changes are recorded,” the error was related to identifying the information obtained through interrogation of the patient and that obtained from the physical examination, with the only difference being where this information was documented within the medical record.

When grouping disagreements by sections of clinical history, a clear gradient emerged: “History of present illness” accounted for the highest number of discrepancies (n=125), followed by “Medical history by organ and system” (n=100), whereas “Medical history and lifestyle” (n=51) and “Physical examination” (n=49) showed substantially lower disagreement rates. The other section did not stand out regarding the number of disagreements.

The “History of present illness” section is inherently less structured and relies on free-text narrative describing the chief complaint and symptom characteristics. Unlike other sections, its content is highly dependent on the specific clinical scenario and on the student’s judgment regarding relevance, leading to variability in both expression and interpretation. Consequently, evaluators must interpret heterogeneous and often unstructured descriptions, increasing the likelihood of disagreement. A similar, though less pronounced, effect is observed in the “Medical history by organ and system” section, which, despite being more standardized, is frequently adapted to the presenting complaint and partially overlaps with information already described in the previous section. In contrast, “Medical history” and “Physical examination” are highly standardized sections with consistent structure and predictable item-response patterns, facilitating straightforward identification and evaluation of rubric criteria.

### Detailed Analysis by Experts

Because of the high error rates shown by some items and the low value of specificity, an in-depth analysis of the rubrics was carried out using the reasoning provided by CoT. The evaluators reviewed CoT arguments for 536 items (389 FPs and 148 FNs) where their judgment diverged from that of Claude. Discrepancies occurred in the following sections of the clinical record: patient identification and chief complaint (n=2), medical history (n=83), history of present illness (n=331), physical examination (n=77), assessment and plan (n=20), and order and clarity of writing (n=23). The physician evaluators confirmed 342 arguments (63.8% of original errors) in favor of Claude and maintained their own judgment in the remaining 194 arguments (remaining 36.2%). Consequently, the initial accuracy of 85.0% (3046 correct out of 3582 items) increased substantially after revision, yielding a revised value of 94.6% (SD 4.3%), representing an improvement of approximately 9% over the original evaluation.

In terms of sensitivity and specificity, there was also a notable improvement. FPs reduced from 389 to 142, and FNs reduced from 148 to 52. Sensitivity increased by 3.8%, rising from 94.2% (SD 5.9%) to 98.0% (SD 2.3%), indicating enhanced detection of valid corrections. More prominently, specificity improved by 23%, increasing from 60.3% (SD 18.2%) to 83.3% (SD 14.3%), reflecting a substantial reduction in FPs and a greater reliability in rejecting invalid corrections. The large SD indicates notable interevaluator variability, implying that evaluators applied heterogeneous criteria when rejecting invalid corrections.

Considering the information from Figure 4, we compiled the number of FPs and FNs of outstanding errors before and after the corrections for Claude. This information has been compiled in Table 5.

**Table 5.** FP^a^ and FN^b^ distributions per item after educators’ correction.

Item	FP before, n	FP after, n	FP improvement (%)	FN before, n	FN after, n	FN improvement (%)
04	1	0	100	14	9	35.7
06	13	6	53.8	0	0	—^c^
19	13	7	56.6	0	0	—
21	20	7	33.3	4	0	100
26	24	10	58.3	4	0	100
28	23	8	65.2	0	0	—
32	19	13	31.5	1	0	100
44	12	7	41.6	15	10	33.3
48	7	2	71.4	13	12	7.7
49	0	0	—	5	5	0
Total	132	61	53.7^d^	56	36	35.7^e^

a FP: false positive.

b FN: false negative.

c Not applicable.

d μ: 63.5.

e μ: 64.9.

As shown in Table 5, FPs were more frequent than FNs. This finding is understandable given that 72.5% (2599/3582) of items were labeled “Yes.” Moreover, a clear improvement was observed in both FP and FN rates across the evaluated items. Overall, FP values had a 63.5% reduction, while FN values experienced a 64.9% improvement. These results indicate a substantial improvement in precision and sensitivity. In particular, items 4, 28, and 48 showed the highest FP improvements (100.0%, 65.2%, and 71.4%, respectively), while items 21, 26, and 32 achieved complete elimination of FNs (100% improvement). For item 4, which concerns the existence of nondrug allergies, there was only 1 FP, which turned out to be a TP. Items 21, 26, 28, and 32, which can be reviewed in Multimedia Appendix 1, highlight the difficulty for human evaluators to capture subtle information within the narrative of the medical record, which may be contained elsewhere in the description. Item 48, which involves the order and clarity of the medical record, is a global qualitative criterion, and it does not verify the existence of specific information. Only item 49 maintained unchanged FN values.

All these improvements in the metrics demonstrate the added value of incorporating CoT reasoning, which facilitated the identification of evaluator errors and significantly enhanced overall performance.

## Discussion

### Principal Findings

The primary objective of this study was to evaluate whether LLMs could effectively support medical instructors in the assessment of clinical records and whether CoT prompting could enhance this process. The results confirm both hypotheses, with Claude 3.5 Sonnet consistently outperforming Llama 3.1 70B across all metrics and experimental configurations. While CoT and one-shot strategies yielded comparable overall accuracy values for Claude (85.0% vs 86.4%) initially, CoT achieved a markedly superior sensitivity, specificity, precision, and *F*_1_-score, making it the best-performing configuration when a comprehensive evaluation of model behavior is considered.

Sensitivity was uniformly high across all model-strategy combinations, indicating that all configurations reliably detected valid student responses. Claude with CoT achieved the highest sensitivity (mean 98.1%, SD 2.2%), reflecting a strong capacity to minimize FNs and avoid penalizing students for correct answers. This consistency across configurations suggests that the task of identifying mentioned concepts in clinical records is, in general, well-suited to LLM-based evaluation regardless of the specific prompting approach used.

Specificity, by contrast, presented a more nuanced picture. Initial values were notably lower (83.2%), accompanied by high SDs across all configurations, which might initially suggest a systematic limitation of the models. However, closer examination revealed that this variability was largely attributable to inconsistencies in human reference evaluations rather than to model errors per se. This interpretation was confirmed through a structured adjudication process, in which 3 experienced medical professors independently reviewed all discrepant items under partial blinding, consulting the model’s CoT reasoning only as a final reference when disagreement persisted.

Evaluators sided with Claude in 63.8% of the 536 reviewed discrepancies, resulting in a revised accuracy of 94.6% (SD 4.3%), an improvement of approximately 9 percentage points over the initial estimate. FPs reduced from 389 to 142, and FNs reduced from 148 to 52, with sensitivity rising to 98.0% (SD 2.3%), and specificity improving substantially to 83.3% (SD 14.3%). The persistently high SD in specificity reflects genuine interevaluator variability in the application of criteria across different case types, rather than instability in the model itself. Precision and *F*_1_-score also improved, with their updated mean values being 94.6% (SD 5.8%) and 96.2% (SD 3.3%), respectively.

The stratified sensitivity analysis confirmed that model performance was robust across all 4 clinical scenarios (cholecystitis, tensional headache, polymyalgia rheumatica, and mechanical chest pain), with overall type-wise accuracy remaining stable at 94.0% (SD 1.5%) and no substantial degradation observed in any case type. Specificity showed the greatest variability across scenarios (78.7%‐90.8%), with lower values for headache and polymyalgia rheumatica, suggesting that cases with higher proportions of positive labels and more complex clinical narratives are somewhat more prone to FPs.

Claude 3.5 Sonnet achieved a higher number of both TPs and TNs, along with substantially fewer FNs and FPs, compared to Llama 3.1. This translates to both higher sensitivity, reflecting a stronger ability to recognize valid corrections, and higher specificity, indicating improved rejection of invalid corrections. In contrast, Llama exhibited a noticeable increase in FPs, suggesting a tendency to overestimate item correctness. These findings confirm that Claude provides a more balanced and reliable evaluation.

Analysis of error distribution by rubric section revealed a clear gradient, with “History of present illness” accounting for the highest number of discrepancies (n=125), followed by “Review of systems” (n=100), while “Medical history” and “Physical examination” showed substantially lower disagreement rates (n=51 and n=49, respectively). This pattern is consistent with the inherently unstructured nature of the former sections, where free-text narratives and student-dependent judgments introduce greater variability in both expression and interpretation. At the item level, both models exhibited localized difficulties in semiological descriptors, such as symptom characteristics, intensity, associated symptoms, and perceived cause, as well as in items requiring precise localization of information within the record’s structure, such as distinguishing interrogation-derived data from physical examination findings.

Taken together, these findings demonstrate that CoT reasoning provides measurable benefits beyond aggregate accuracy improvement. Its interpretable, step-by-step rationale serves as an auditable record of the evaluation process, enabling the identification of annotation inconsistencies that would otherwise remain hidden. This capacity to surface latent disagreements in human-generated reference labels constitutes one of the most significant contributions of the approach, with direct implications for rubric refinement and grading consistency in future evaluation cycles.

### Comparison With the Literature

Several other studies addressed issues related to those observed in our evaluation. For example, Luordo et al [35] provided a particularly relevant point of reference given its shared linguistic context. The authors evaluated GPT-4 for grading clinical reports in a cardiology Objective Structured Clinical Examination (OSCE) station involving 96 fourth-year medical students, comparing AI scores against 2 expert human graders and 1 inexperienced grader. The results showed a significant overall correlation between AI and expert evaluators (intraclass correlation coefficient [ICC]=0.77 for single measures and ICC=0.91 for average measures); however, the AI system was systematically more stringent, assigning scores on average 3.51 points lower than human experts. This rigidity was attributed to the model’s literal adherence to checklist criteria, which prevented it from accommodating appropriate variation in medical language or implicit clinical inferences. This is a limitation that the authors argue reflects the quality of item design rather than a fundamental flaw in the AI approach.

Additionally, a study [36] compared GPT-4 with a human proctor in grading 127 clinical notes written by third-year medical students following an OSCE, using a zero-shot prompting strategy with a predefined rubric covering 4 categories: history, physical examination, differential diagnosis, and treatment plan. While the mean total score differed by only 1 point (3.5%), ChatGPT-4 assigned significantly more honors grades than the human proctor (92.9% vs 63.8%), with the largest discrepancy observed in the treatment plan section (Cohen *d*=1.25). Crucially, a subanalysis yielded *R*² values between 0.01 and 0.098, indicating that AI scores were not meaningfully aligned with human evaluative judgments at the individual level. Consistent with our findings, the history and physical examination sections showed the lowest disagreement rates, suggesting that structured and objective sections of clinical documentation are more amenable to automated evaluation. The authors concluded that zero-shot LLM grading is better suited for formative rather than summative assessment, a recommendation that aligns with our use of CoT reasoning as a support tool for instructors rather than a replacement for expert judgment.

On the question of human oversight, another study [35] has advocated for a hybrid model in which AI provides an initial grading layer, while a qualified human grader reviews and finalizes scores, in alignment with the human-in-the-loop requirements outlined in Article 14 of the European Union AI Act for high-risk AI systems [37]. Our study operationalizes a methodologically rigorous version of this model. Rather than using human oversight solely as a corrective safety net, we implemented a structured adjudication protocol in which 3 experienced medical professors independently reanalyzed all discrepant items under partial blinding, resorting to the model’s CoT reasoning only as a final reference when disagreement persisted. This approach served a dual function: it preserved the epistemic authority of clinical expertise while systematically using the model’s reasoning to challenge and refine human judgment. The result was not merely an improvement in performance metrics but a substantive contribution to rubric quality that is expected to benefit future evaluation cycles. Taken together, these comparative findings suggest that the most productive role for LLMs in clinical education assessment is not as autonomous graders but as structured reasoning partners that enhance the reflective capacity of human evaluators and expose the hidden biases in the criteria they apply.

### Implications of the Findings

In our study, we compared the evaluations performed by the LLMs with those of a human assessor and reviewed all items showing discrepancies, achieving a coincidence rate of 94.6%. According to the literature, the minimum acceptable level of interrater reliability is 60%, with 80% considered the gold standard [14].

The use of LLMs combined with CoT reasoning may offer multiple advantages, not only in terms of resource efficiency but also by serving as a second evaluator alongside a human rater, thereby reducing grading errors and providing valuable feedback through CoT-based comments, which can enhance students’ learning progression.

In our study, we examined interrater agreement across all records in which discrepancies were identified and found that disagreements favored AI in 63.8% of cases. In other studies, such as the study by Jamieson et al [23], where only half of that proportion favored AI, this difference could be explained by the fact that, unlike our work, only 17% of the records (those corresponding to students with the lowest scores) were reviewed.

Our findings support the growing body of evidence suggesting that the use of AI in medical education provides advantages in terms of standardization, objectivity, and immediate feedback. However, since our study focused exclusively on the evaluation of clinical records, we did not assess other critical aspects of the clinical encounter, such as contextual case interpretation or interpersonal communication, where recent studies have identified notable limitations.

### Strengths and Limitations

The main strength of our study is the CoT-based approach because of its high interpretability level when compared to its alternatives. It allowed us to identify human-made mistakes in the original labels and provided insights into the evaluation process. The outputs of most classification models cannot be easily explained. Moreover, neural network–based approaches are commonly known for being “black boxes.” This problem gets even worse when the parameter count scales up, as the model outputs become harder to trace through the network.

On the other hand, rule-based classification models offer better interpretability, but lack the required power for natural language processing tasks.

Our approach offers a good balance in between, as it allows easy audit of the results by eliciting the evaluation process in a structured way, and the implicit reasoning generally provides better accuracy, as shown by the current test-time-compute–based trends. It also does not require parameter tuning, making the adaptation process simpler and cheaper than LoRA or reinforcement learning approaches.

Another strength is the possibility of offering teachers an evaluation support tool that will allow them to give detailed feedback to students and to be able to improve the number of student evaluations during their training.

Despite the satisfactory outcomes, several limitations should be considered when interpreting the findings of this study.

First, the dataset analyzed in this study was relatively limited in size, as only 79 clinical records were evaluated. It also had limited diversity. Although the dataset included multiple clinical scenarios (polymyalgia rheumatica, headache, acute cholecystitis, and chest pain) and several levels of student performance, the results may not fully generalize to other institutions, languages, clinical contexts, or assessment frameworks.

Second, the reference scores used as the ground truth correspond to the evaluations assigned before the study rather than to a reference standard specifically validated for this experiment. Consequently, some degree of subjectivity in the reference evaluations cannot be completely excluded, which may influence the interpretation of the reported performance metrics.

Third, occasional API request interruptions occurred during batch processing due to temporary usage quota limits. These failures affected requests randomly and independently of the content of the clinical records, and therefore, they did not introduce systematic bias into the evaluation process, although they slightly reduced the total number of processed items. The final dataset of 3582 Claude-processed items is therefore considered representative and valid for the reported analyses.

### Conclusions

This study aimed to evaluate the benefits of CoT and LLMs for the task of correcting clinical records written in Spanish by undergraduate students. For this purpose, we initially compared the performance of this strategy against a well-known strategy (one-shot). The strategy was applied to Llama 3.1 and Claude 3.5, which corrected 79 clinical histories with 48 or 49 rubric items. In the first approach, the strategy yielded strong results in all metrics, except for specificity, which underperformed in all combinations of strategies and models, suggesting that some items were corrected wrongly. The use of CoT reasoning proved beneficial, as it allowed us to identify which corrections made by the evaluators had been processed incorrectly. This approach has been shown to consistently reduce FNs and FPs in the models evaluated, especially in items with negations or indirect mentions, and provides traceability (the exact fragment is highlighted) and reviewable quality for the clinical team. In practice, CoT not only improves the detection of difficult concepts but also enables the identification and correction of discrepancies in human annotations during validation. After benefiting from CoT insights, our model obtained an accuracy of 94.6%, specificity of 83.3%, and sensitivity of 98.0%. The persistently high SD in sensitivity indicates substantial variability among evaluators, suggesting that they applied differing criteria when identifying invalid corrections.

Future studies should replicate the analysis with larger and more diverse datasets across multiple medical schools to enhance the external validity and generalizability of the findings. Incorporating institutions with different curricula, assessment formats, and student populations would allow for a more robust evaluation of the model’s performance under heterogeneous educational conditions. Additionally, expanding the datasets to include a broader range of question types, difficulty levels, and clinical domains would help assess the model’s consistency and reliability across varied learning scenarios. Such efforts would also enable the analysis of potential biases and support the development of more equitable and adaptable AI-driven assessment systems. Finally, future work could explore prompt tuning techniques, such as GEPA [38], to further enhance accuracy without high computational costs.

## Supplementary material

10.2196/88652 Multimedia Appendix 1 Example of the rubric: clinical case of chest pain.

10.2196/88652 Multimedia Appendix 2 Example of a prompt sent to the large language models using chain-of-thought.

## Abbreviations

AI: artificial intelligence
API: application programming interface
CoT: chain-of-thought
FN: false negative
FP: false positive
ICC: intraclass correlation coefficient
LLM: large language model
LoRA: low-rank adaptation
OSCE: Objective Structured Clinical Examination
TN: true negative
TP: true positive
