# Supplementary material for: Benefits of Chain-of-Thought Prompting for Clinical Record Rubric Evaluation in Undergraduate Medical Education: Experimental Evaluation Study With Medical Faculty
Source: JMIR Med Educ. 2026 Jul 23;12:e88652. doi: 10.2196/88652 (PMC13395432; doi:10.2196/88652)
Supplement: Multimedia Appendix 1 [file mededu-v12-e88652-s001.docx]

Appendix 1 - Example of rubric: Clinical case of chest pain

1. **PATIENT IDENTIFICATION AND CHIEF COMPLAINT**
2. Patient name and age are documented
3. Chief complaint (CC) is documented at the beginning of the report
4. **PAST MEDICAL HISTORY (PMH)**
5. Allergies and/or adverse drug reactions are recorded
6. Allergies and/or intolerances (food, pollen, iodine) are documented
7. Personal history of Hypertension (HTN), Diabetes Mellitus (DM), Dyslipidemia (DL) is documented
8. Other relevant personal history (cancer, cardiovascular disease CVD…) is included
9. Surgical history is recorded
10. If female: Obstetric and gynecological history (menstrual cycle, GPA) is documented
11. Family history of HTN, DM, DL, CVD, cancer is recorded
12. Alcohol, tobacco, and other drug use are documented
13. Treatment: medications or herbal products (dose and regimen) are listed
14. Family situation (single, partnered, separated, children, cohabitants) is described
15. Employment status is documented
16. Dietary habits are recorded
17. Physical activity is documented
18. Vaccination status is included
19. **HISTORY OF PRESENT ILLNESS (HPI)**
20. Reason for the visit is documented
21. Onset/duration is recorded ("Since when")
22. Symptom location (frontal, occipital, unilateral, bilateral) is specified
23. Pain radiation is documented
24. Symptom characteristics (pressing, stabbing, pulsatile, etc.) are described
25. Symptom intensity is recorded
26. Aggravating or alleviating factors are documented
27. Functional limitation it causes is described
28. Any medication taken for the symptom is documented
29. Associated symptoms are described
30. Perceived cause of the symptoms is documented
31. Previous episodes of similar symptoms are recorded
32. **REVIEW OF SYSTEMS (ROS)**
33. General symptoms: asthenia, anorexia, weight loss, fever are documented
34. Neurological symptoms: headaches and sensory changes (vision, hearing, etc.) are recorded
35. Mental health: mood, insomnia are documented
36. Respiratory symptoms: cough, expectoration, dyspnea are recorded
37. Cardiovascular symptoms: chest pain, orthopnea, edema are documented
38. Digestive symptoms: dysphagia, dyspepsia (heartburn, abdominal pain), and bowel habits (frequency, stool characteristics) are recorded
39. Genitourinary symptoms: dysuria, nocturia, hematuria, menstrual problems are documented
40. Musculoskeletal symptoms are recorded
41. Skin and adnexal symptoms are documented
42. **PHYSICAL EXAMINATION (PE)**
43. Vitals: BP, HR, RR, O₂ saturation, temperature, blood glucose (if applicable) are recorded
44. General condition: good general condition (GGC)/ poor general condition (PGC), hydration, color are documented
45. Head and neck: pupils, lymph nodes are examined and documented
46. Cardiac auscultation: frequency, rhythm, presence of murmurs are recorded
47. Pulmonary auscultation: clear to auscultation bilaterally (CTAB) or additional sounds are documented
48. Abdomen: bowel sounds, palpation (soft, depressible), any pain on palpation; other signs if applicable are documented
49. Lower extremities (LE): presence or absence of edema, color changes are recorded
50. **ASSESSMENT AND PLAN**
51. Complementary tests are ordered
52. Clinical judgment or differential diagnosis is established
53. Therapeutic plan for the patient is proposed
54. **ORDER**
55. Clinical history is organized and understandable
