# Supplementary material for: Benefits of Chain-of-Thought Prompting for Clinical Record Rubric Evaluation in Undergraduate Medical Education: Experimental Evaluation Study With Medical Faculty
Source: JMIR Med Educ. 2026 Jul 23;12:e88652. doi: 10.2196/88652 (PMC13395432; doi:10.2196/88652)
Supplement: Multimedia Appendix 2 [file mededu-v12-e88652-s002.docx]

Appendix 2 - Example of a prompt send to the LLMs using CoT

Your task is to determine whether a specific medical concept IS MENTIONED in a clinical record. IMPORTANT: The presence of the concept must be recorded as "YES" whether it is mentioned positively or negatively, including when its existence is denied or ruled out.

Concept to search: {item}

Follow these analysis steps:

1. Analyze the text systematically:
2. Look for direct mentions of the exact concept.
3. Identify variations of the concept:

- Technical medical synonyms
- Equivalent colloquial terms
- Related medical abbreviations
- Grammatical or wording variations

1. Detect the context of the mention:

- Direct statements
- Negations or exclusions
- Indirect references

1. Verify that a negation ("does not present", "no evidence of", "is ruled out") counts as a MENTION of the concept.
2. Review the entire text with the following criteria:
3. A symptom (patient’s description) is not the same as a sign (medical observation).
4. If the physician has not written anything about the concept, it is not considered a mention.
5. If the concept is mentioned in a family member but not in the patient, it is not considered a mention.
6. The concept must appear completely or partially, but in a recognisable and exact way. Vague or ambiguous mentions are not considered valid.
7. The conclusion YES or NOT must be consistent with the detailed analysis.

Your response must follow EXACTLY this format:

<analysis>

DIRECT_MENTION: YES/NO

SYNONYMS_FOUND: COMMA_SEPARATED_LIST

CONTEXT: POSITIVE/NEGATIVE/MIXED/NOT_APPLICABLE

EXCERPT: EXACT_TEXT_WHERE_IT_APPEARS/DOES_NOT_APPEAR

REASONING: BRIEF_EXPLANATION_OF_THE_DECISION

CONCLUSION: YES/NO

</analysis>

Return only the analysis block with the tags <analysis> and </analysis>.

Example:
Concept: "Psychiatric or psychological disorders"

Text: "... (does not appear) ..."

<analysis>

DIRECT_MENTION: NO

SYNONYMS_FOUND: mental illnesses

CONTEXT: NOT_APPLICABLE

EXCERPT: DOES_NOT_APPEAR

REASONING: The concept is not mentioned, either directly or indirectly. CONCLUSION: NO

</analysis>

This is the current evaluation rubric:

<rubric>

{rubric}
</rubric>

Notes on the rubric (apply only to the related points):

{medical_texts}

Now, please analyze the following text for the concept "{point}", ensuring that you ONLY perform the analysis for the requested concept and not for other concepts that may appear in the rubric:

<record>

{record}

</record>
